# Novel Twenty20 batting simulations: a strategy for research and improved practice

**DOI:** 10.12688/f1000research.52783.1

**Published:** 2021-05-21

**Authors:** Tiago Lopes, David Goble, Benita Olivier, Samantha Kerr

**Affiliations:** 1 Physiology, University of the Witwatersrand, Johannesburg, Gauteng, 2193, South Africa

**Keywords:** Cricket, batting performance, simulation, innings

## Abstract

Twenty20 cricket and batting in particular have remained vastly understudied to date. To elucidate the effects of batting on the batter, tools which replicate match play in controlled environments are essential. This study describes the development of two Twenty20 batting simulations, for a high and low strike rate innings, generated from retrospective analysis of international and domestic competition. Per delivery analysis of probabilities of run-type and on/off-strike denomination produce ball-by-ball simulations most congruent with retrospective competitive innings. Furthermore, both simulations are matched for duration and dictated through audio files. The `high' strike rate innings requires a batter to score 88 runs from 51 deliveries, whereby 60 runs are from boundaries. Similarly, the `low' strike rate innings requires a batter to score 61 runs from 51 deliveries, where 27 runs are scored from boundaries. Because batting simulations dictate run scoring outcomes, a method of quantifying a batter's performance from bat-ball contact scores is described. Ten elite batters achieved a mean performance score of 72 (SD = 26) and 88 (21) for the low and high strike rate simulations respectively. This study provides sport practitioners with a training technique to improve specific skill acquisition and enables research in understudied Twenty20 batting.

## 1 Introduction

Batting in the sport of cricket is an interceptive task which creates a combination of physical ( Johnstone & Ford, 2010; Pote & Christie, 2016) and cognitive stress ( Goble & Christie, 2017). In a cricket match, runs are scored by sprinting between the wickets or by hitting the ball out of play (boundaries). During an innings, a batter and batting partner will attempt to accumulate runs, while trying to avoid being dismissed. Resultantly, a batter’s innings may be prolonged resulting in fatigue ( Christie, Todd, & King, 2008; Johnstone & Ford, 2010) which may adversely affect batting performance. Research has suggested traditional net-practice may be inadequate to replicate the demands of an innings produced in competitive match-play ( Goble & Christie, 2017; Vickery et al., 2018) and thus batters may not experience batting under appropriate fatigue, which may limit the effectiveness of training. Woods and colleagues (2019) further highlighted the value of practice which attempts to closely replicate match-play for the development of skill. The availability of reliable match data in cricket enables the development of detailed batting simulations which may be invaluable tools in practice programmes and research investigating the physiology of a batter.

In previous studies, attempts to replicate a batting innings have taken various forms. Christie and colleagues (2008) replicated the physical demands of batting through shuttle running. Running multiple shuttles, the length of a cricket pitch (17.68m), physically mimics run scoring in cricket. However, shuttle running protocols neglect to account for the energetic cost of ball striking and boundaries where no shuttles are required. As a result, more sophisticated protocols which require batters to intercept deliveries were subsequently developed. The Battlezone protocol mimics a small-sided cricket match reduced to eight players and six repeated bouts, each consisting of eight overs ( Vickery, Dascombe, Duffield, Kellett, & Portus, 2013). Batters are required to compete in a bout against two bowlers and four fielders in a 27m ring. However, the limited length (six overs per batting pair) and smaller field may limit the ecological validity of this protocol and therefore its use in fields of research and elite sport. Finally, the BATEX ( Houghton, Dawson, Rubenson, & Tobin, 2011) protocol simulates scoring a One Day International (ODI) century (100 runs) with ball-by-ball audio guidance. In this protocol batters are required to complete an innings of two hours and 20 minutes, the typical length of an ODI century ( Houghton et al., 2011). Unlike the Battlezone protocol, which provides batters with freedom to score runs at any rate, BATEX instructs batters on the denomination of runs required – singles, doubles or boundaries - every over. Thus, BATEX mimics the amount of time spent at the crease and also allows the innings to follow a run scoring progression akin to centuries scored in ODI’s. Further, the BATEX protocol is divided into phases which allows researchers and sport practitioners to manipulate scoring zones and running intensities throughout the innings which adds a form of ecological dynamics and match realism suggested by Woods and colleagues (2020; 2019). However, the BATEX protocol, being a simulation of the ODI format, may not be applicable to the increasingly popular, shorter Twenty20 format where an entire team innings is often completed within two hours.

To date no batting protocol has specifically aimed to address a Twenty20 innings. Batting may be performed at various run scoring intensities (strike rate) depending on the context of the innings and format of the game. Twenty20 cricket is the shortest format of the game (20 overs per side) resulting in higher run scoring intensities as batters attempt to score more runs from fewer available deliveries. Thus, batting simulations which are unique to Twenty20 cricket are required. Further, the BATEX protocol places minimal emphasis on the role of the batter’s partner. As batting is performed in pairs, consideration for how runs are scored while the batter in question is off-strike is necessary as this increases the workload placed on the batter.

This study aimed to develop two Twenty20 batting simulations which mimic the demands of competitive match play. To further highlight the impact of strike-rate, both simulations possess an equal number of deliveries, resulting in varying run scoring intensities throughout the innings.

## 2 Methods

**Figure 1.**
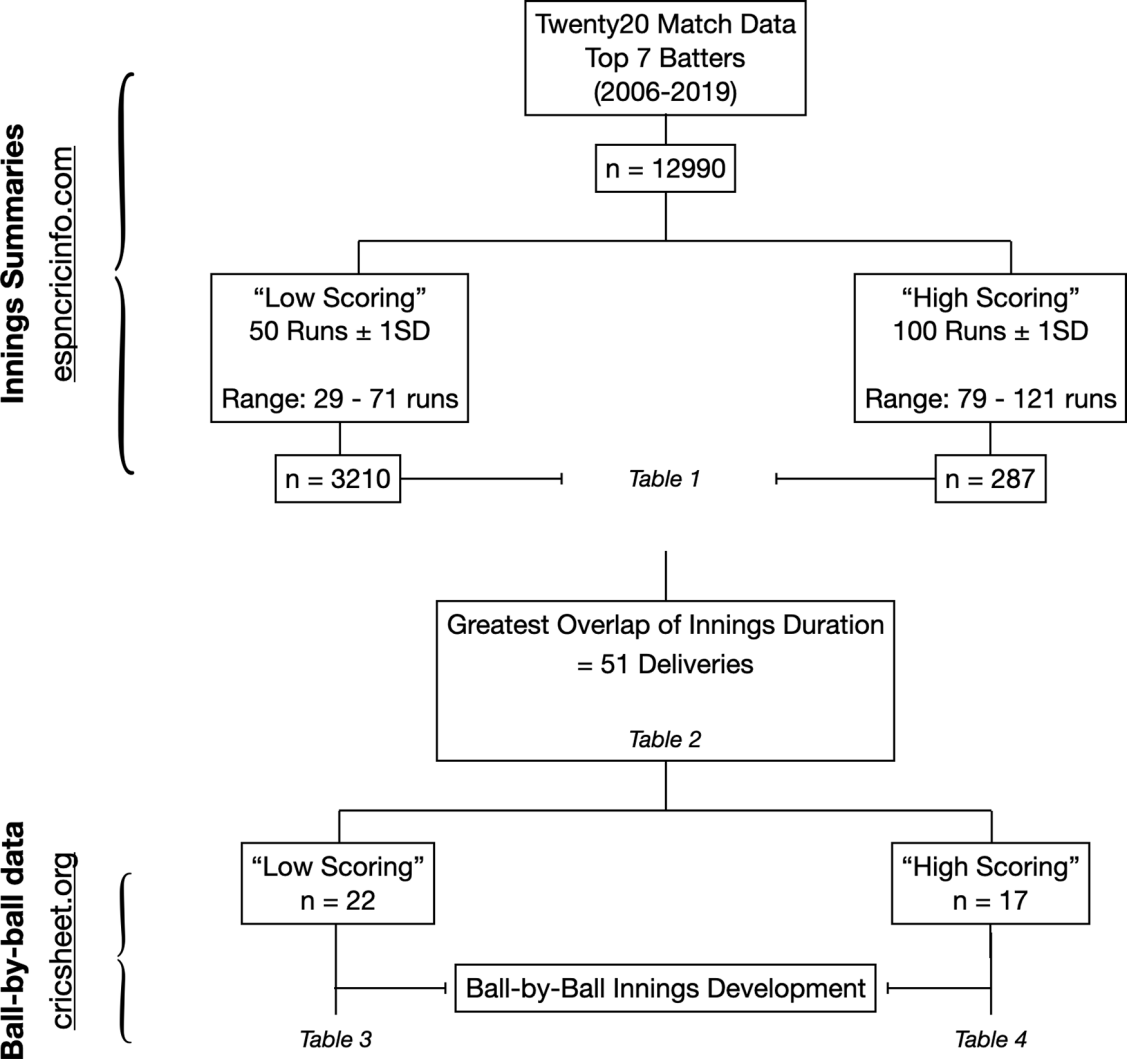
Schematic outline of data reduction procedures and relevant innings selection from online databases. Table 1 provides innings summaries of specialist batters within each ‘low’ and ‘high’ scoring subcategory. Table 2 provides refined innings summaries from each ‘low’ and ‘high’ subcategory with an equal number of on-strike deliveries. Table 3 and Table 4 represent the developed ball-by-ball simulations.

Two Twenty20 simulations were developed to simulate match play for an individual achieving a 1) ‘high’ and 2) ‘low’ strike rate innings. Both high and low strike rate simulations are equal in duration, achieved by standardizing the number of deliveries in both simulations. However, the strike rate and mode of run scoring in either innings varies in accordance with data obtained from selected innings. Relevant retrospective innings selection procedures and accessed databases are outlined in Figure 1.

### 2.1 Retrospective data acquisition - innings summaries

Match data from previous Twenty20 internationals (2006–2019) and the domestic Vivo Indian Premier League (IPL) competition (2008–2019) were exported from ESPNcricinfo ( ESPN Sports Media Ltd, n.d.) using an Excel macro. ESPN’s statistical database ‘Statsguru’ provided match summaries and run tallies of individual innings produced in international competition. An advanced filter was applied to query an innings by innings list of top order batters (top seven) from all available seasons and competitions. To obtain innings summaries from the IPL, match scorecards were opened manually to import summaries produced the by top seven batters from both teams. This process was repeated for every fixture within a season and from all available seasons at the time of analysis. Thus, a total sample of 12,990 individual innings were selected. These innings represented 5906 different batters, runs mean = 19.9 (SD = 21.2).

Two batting milestones, a half-century (50 runs) and century (100 runs), were selected to represent a ‘low’ and ‘high’ strike rate threshold. These milestones were selected as they represent meaningful team contributions and, secondly, are likely to be of a duration which warrants physiological study. From the 12,990 innings, 87 innings produced exactly 50 runs scored and only nine innings matched the 100 run criteria. Therefore, to increase the sample sizes around the chosen milestones, a range of ±1 standard deviation of runs scored (SD = 21.2 runs) was applied. Thus, the half century (low strike rate) sample size increased to n = 3210 and included innings of 29-71 runs scored. Likewise, the century (high strike rate) sample size increased to include innings ranging from 79-121 runs scored, producing an n = 287. The innings summaries for these adjusted milestones are presented in Table 1.

**Table 1.** Average characteristics of individual innings milestones broadened by ± 1SD of runs scored.

	‘low’ strike rate (50 runs ± 1SD)				‘high’ strike rate (100 runs ± 1SD)			
	Mean	(SD)	Median	Range	Mean	(SD)	Median	Range
Runs scored	44.2	(11.5)	42	29–71	91.1	(10.3)	89	79–119
On-strike deliveries	31.8	(9.9)	31	8–79	52.9	(8.6)	53	25–119
Minutes at the crease	48	(17)	46	7–109	78	(14)	81	37–109
Fours ^a^	4.1	(2.1)	4	0–12	8.5	(2.8)	8	1–16
Sixes ^b^	1.7	(1.4)	1	0–9	4.7	(2.5)	5	0–13
Strike rate ^c^	147	(39)	139	67–422	177	(30)	171	123–194

Data represent competitive individual innings produced in Twenty20 internationals and the Vivo Indian Premier League between 2006–2019. Innings milestones are broadened to categorize a range of ± 1SD (21 runs) around the given milestone, increasing the number of innings within each category. ‘low’ strike rate n = 3210 individual innings, ‘high’ strike rate n = 287.

^a^ Intercepted delivery which leaves the field of play with a minimum of one bounce, batter credited four runs.

^b^ Intercepted delivery which leaves the field of play without a bounce, batter credited 6 runs.

^c^ Run scoring intensity - fraction of runs scored and on-strike deliveries.

The number of on-strike deliveries was selected as a robust measure of innings length. To standardize the duration of both batting simulations, frequency distributions of deliveries faced within each innings subset were drawn and the point of intersection with the largest sample size between the ‘high’ and ‘low’ strike rate innings was identified. The resultant overlap occurred at 51 deliveries faced, with a total of 27 half-centuries (‘low’) and 18 centuries (‘high’). While these innings were of equal duration, it is important to note that fundamental differences in runs scored, strike rate, number of boundaries and ratio of boundaries to runs accumulated by running - define the intensity of each simulation. Therefore, to account for each of these factors and to develop accurate simulations, ball-by-ball match data was needed to provide records of how each individual innings (‘low’, n = 27 and ‘high’, n = 18) progressed and how runs were accumulated throughout the innings.

### 2.2 Innings development - ball-by-ball match data

Ball-by-ball data was obtained from a second online database ( Rushe, 2009). However, at the time of analysis, existing ball-by-ball match data from the online database reduced our sample to 22 ‘low’ and 17 ‘high’ strike rate innings - presented in Table 2.

**Table 2.** Average characteristics of broadened innings milestones at the point of greatest overlap in number of on-strike deliveries.

	‘low’ strike rate (n = 22)				‘high’ strike rate (n = 17)			
	Mean	(SD)	Median	Range	Mean	(SD)	Median	Range
Runs scored	61.3	(8.9)	65	39–71	90.4	(11.1)	89	79–117
Minutes at the crease	61	(14)	60	51–101	76	(14)	81	55–112
Fours ^a^	5.4	(2.4)	6	1–10	8.5	(2.9)	9	4–15
Sixes ^b^	1.8	(1.5)	1	0–5	4.4	(2.9)	4	1–13
Strike rate ^c^	120	(18)	128	76–139	177	(22)	175	154–229

Data represent competitive individual innings produced in Twenty20 internationals and the Vivo Indian Premier League between 2006–2019 with a standardized workload of 51 on-strike deliveries.

^a^ Intercepted delivery which leaves the field of play with a minimum of one bounce, batter credited four runs.

^b^ Intercepted delivery which leaves the field of play without a bounce, batter credited 6 runs.

^c^ Run scoring intensity - fraction of runs scored and on-strike deliveries.

Match data was downloaded as comma separated variables (CSV’s) and reduced to isolate the relevant innings within the match (Lopes, 2021). These CSV’s were imported into an R script for analysis. The purpose of analysing ball-by-ball innings data was to determine; i) the deliveries with the highest frequency of dot balls, singles, twos, threes, fours and sixes, ii) how runs were accumulated, and iii) the scoring rates of each individual innings. Furthermore, because batting is performed in pairs, each innings was analysed for its respective partnership data to determine; i) the average partnership deliveries faced and hence the overall duration of the simulation, ii) the number of deliveries which the batter is most likely on-/off-strike, and iii) the proportion of runs scored by the batter’s partner. By analysing ball-by-ball data, the average number of dot balls, singles, twos, threes, fours and sixes were calculated for both the ‘low’ and ‘high’ strike rate innings.

For each individual innings, the role of the batter’s partner was determined using a second R script. Ball-by-ball partnership data was extracted from the match data for every delivery faced by the batter’s partner/s (Lopes, 2021). This provided the number of deliveries faced during the innings as well as the sequence of runs scored by the batter’s partner/s. Like the development of the high and low scoring innings, the length of the overall partnership (deliveries faced by both batter and partner/s) was determined by the point of greatest overlap in overall partnership length from the representative high and low scoring innings. However, three overlaps were identified each with a sample size of one. As a result, the overlap which was nearest to the median of deliveries faced for the overall partnership was chosen as the best representative of the partner’s role in both high and low strike rate simulations. This overlap was identified as 43 deliveries resulting in a total simulation length of 94 deliveries. To determine which of the total 94 deliveries the batter is on strike for, a strike score was calculated for every delivery by assigning a ‘1’ if the batter was on-strike and ‘0’ if the batter was off-strike for every innings. Thereafter, each delivery was scored using Equation 1.

$$\left(\frac{Ball\;No.}{P.\;Length}\right)\times Strike\;Score$$ (1)

Whereby, ‘Ball No.’ is the sequenced number of the delivery in question (e.g. one, two,… 94), ‘P. Length’ denotes the partnership length or number of deliveries within the given partnership and ‘Strike Score’ denotes the assigned binary score given to the delivery whether the batter was on-strike (1) or off-strike (0). The median was calculated for each delivery and rounded up to identify each ball number (of all 94 deliveries) the batter will be on strike for. This procedure was completed for both simulations.

Once on-/off-strike deliveries were determined, assigning runs to deliveries completed the development of the simulations. For on-strike deliveries, the number of dot balls, singles, twos, threes, fours and sixes were tallied for every delivery (independently for high scoring and low scoring simulations) to determine which deliveries produce the highest frequency/probability of each possible score. From these probabilities run types (dot ball, one, two… etc.) were assigned in descending order of probability. In the case where deliveries produced identical probabilities for two or more run types, priority was given to the run type with the least overall frequency in the innings (for example, sixes were prioritized over dot balls). Lastly, because dictating the exact outcome of each delivery is not representative of match play, every six deliveries (one over) required run types were grouped, giving batters freedom to decide which on-strike delivery, within an over, will produce a given run type according to the stroke played. As studying effects of batting partner/s is not a primary outcome of the batting simulations, required runs from off-strike deliveries were made identical for both high and low scoring simulations. Using the same method described for on-strike deliveries, run type was assigned to each off-strike delivery.

### 2.3 Improving ecological validity

Time motion analysis of Vivo IPL Twenty20 matches was performed and determined the average interval between deliveries to be 35 seconds and 80 seconds between overs, consistent with findings of Houghton and colleagues (2011). An audio recording was created using a written script and computer text-speech function (Supplementary Material A, B) to govern intervals between overs/deliveries. Further, the audio recording describes; i) when the batter is on-/off-strike ii), the run requirements for each over and their denomination (singles, doubles, boundaries etc.), and iii) an indication of the start and end of each over.

During each on-strike delivery, the batter is free to select which on-strike deliveries to score from and what run type is achieved from a given delivery. The only constraints placed are that the batter must match the run type with the achieved stroke and that all required shuttles for an over must be achieved. To match run type with achieved stroke for a given delivery, batters are required to penetrate a scoring zone to be awarded ‘four’ and bat-ball contact must be achieved for all other run types. However, if the number of on-strike deliveries remaining in an over are near-depleted, shuttles may be enforced regardless of achieved contact or scoring zone penetration. For a ‘1’ batters should complete a single shuttle (17.68m) likewise, for ‘2’s batters should complete a double shuttle, and three shuttles for a ‘3’. For all shuttles, batters are required to complete a 180º turn at the end of the crease when completing the final shuttle for a given delivery. Boundaries will require one and a half shuttles for a ‘four’ and no shuttles for a ‘six’. When the batter is off-strike, instruction from the audio tape will dictate the required shuttles necessary for each off-strike delivery and the batter will complete these shuttles beginning on the audio cue. After necessary shuttles have been completed the batter will walk back to the striking crease. Shuttles must be run at maximum intensity akin to match play where batters attempt to maximize the number of runs scored from every delivery.

To improve the ecological validity and dynamics of the simulations, three phases were introduced to represent how an innings would develop under match conditions. These consisted of a ‘PowerPlay’ phase of six overs and two phases of five overs each, and the ’Middle’ and ‘Close Innings’ phases. During these phases, fielding positions or scoring zones can be altered to present varying scoring zones to encourage the batter to manipulate the ball into different areas of the field/arena. In cricket, the Powerplay consists of six overs whereby a maximum of two fielders are placed outside the inner ring while the remaining overs allow a maximum of five fielders outside the inner ring ( ICC, n.d.). No more than five fielders may be placed on the ‘leg-side’ throughout the innings ( ICC, n.d.). Therefore, we suggest a reduction in the number of available scoring zones following the ‘PowerPlay’ phase and discretion in the remaining number and placement of zones depending on the desired objectives of the simulation and skill of the batter.

To incentivise the batter and provide a form of match pressure, an overall batting performance score can be obtained according to the method of Muller and Abernethy (2008). Briefly, the method entails tallying bat-ball contacts according to three criteria. Good ball contact requires contact on the face of the bat and the ball deflecting in the intended direction (2 points). Bad ball contact entails contact with the bat and the ball deflects into an unintended direction (1 point). One point is deducted for an unsuccessful attempt at contact (-1 point) and a `leave' entails an intentional lack of contact with the ball (1 point). The achieved score for a given delivery is doubled if the stroke penetrates a scoring zone. If a batter is dismissed, the final achieved batting score is halved, with further deduction factors applied for multiple dismissals (i.e. two dismissals; performance score divided by three). Batters are instructed to bat for a maximized performance score by ensuring optimal bat-ball contacts, manipulating deliveries into pre-assigned scoring zones as often as possible and avoiding a loss of wicket. Attempting to achieve a maximal performance score in the simulation places a form of pressure on the batter improving match realism. According to the batting performance score criteria, a maximum score of 4 points may be achieved per on-strike delivery resulting in a maximum simulation score of 204 points from 51 on-strike deliveries.

**Figure 2.**
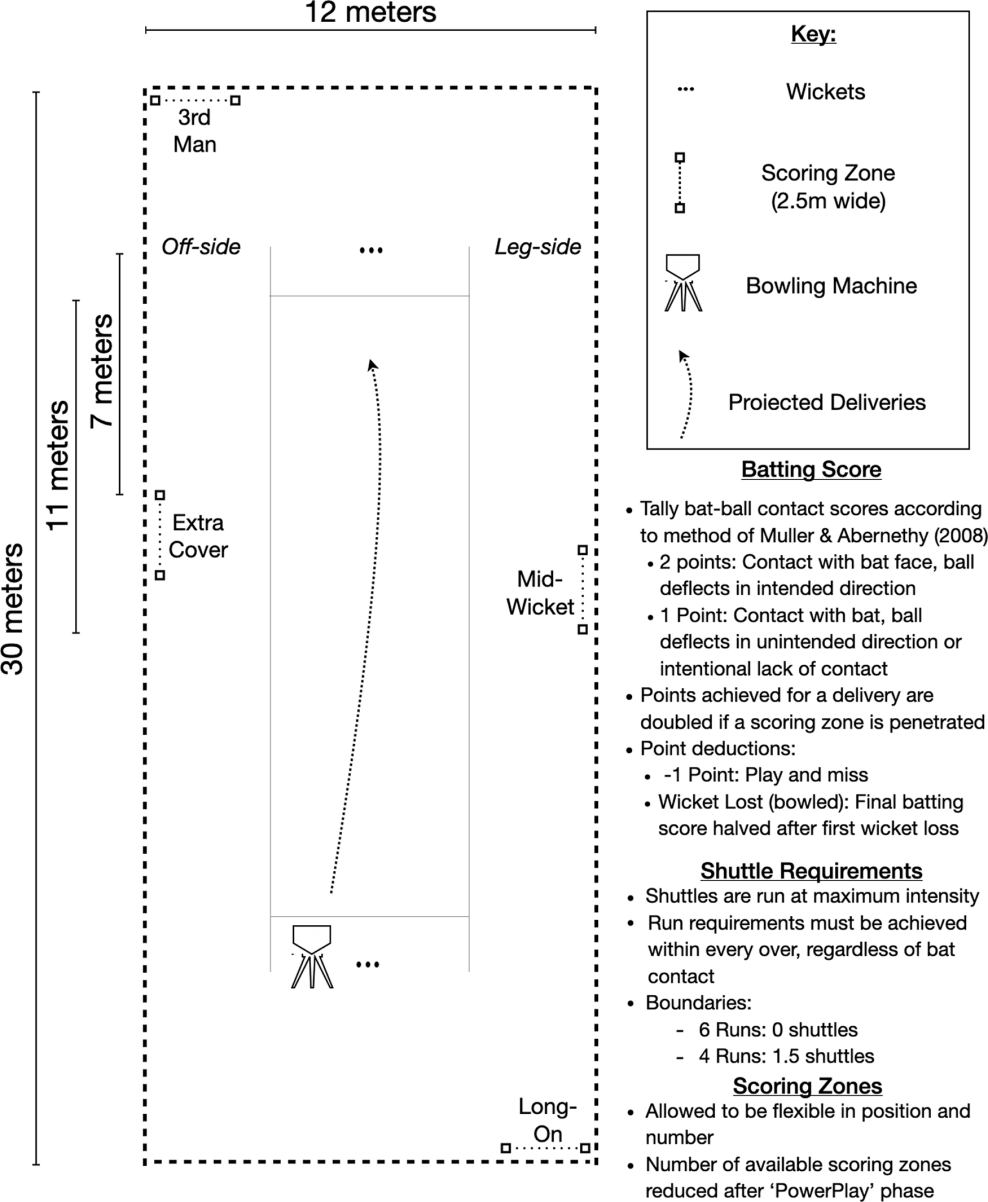
Procedural outline and arena schematic (drawn to scale) depicting regulation size action/indoor cricket arena and regulation length pitch (17.68m).

The method was piloted in a controlled study and recruited 10 experienced (competitive playing experience >6 years) batters who bat in the top seven of their respective teams. Participants were required to be currently competing in amateur cricket leagues and free from injury (age: 22.3 (SD = 3.4) years, stature: 178 (8) centimetres, mass: 80.9 (9.0) kilograms). Procedures were reviewed by the Human Research Ethics Committee (clearance no: M180872) and participants signed informed consent. An arena schematic and procedural outlines are provided in Figure 2. Participants completed both batting simulations, in a randomised order, separated by a minimum of 48 hours. A regulation size indoor/action cricket arena with artificial playing surface was used and deliveries projected from an electric bowling machine Figure 2. In the PowerPlay phase, four scoring zones (2.5 meters wide) were placed in positions to mimic fielding gaps at third-man, extra cover, mid-wicket and long-on. Scoring zones were demarcated with coloured cones and batters were informed to their placement and changes throughout the simulation. Once the PowerPlay phase was concluded, one scoring zone (third-man) was removed to increase the difficulty of achieving boundaries in the ‘Middle’ and ‘Close Innings’ phase.

## 3 Results

The developed simulations are 1 hr 2 min in duration and provided in audio format for ease of accessibility (Lopes, 2021). Ball-by-ball run requirements and on-strike delivery numbers, within an over, are presented in Table 3 (‘low’ strike rate innings) and Table 4 (‘high’ strike rate innings). The low strike rate simulation requires a batter to score 61 runs at a strike rate of 120, whereby 27 runs are scored through boundaries. The ‘high’ strike rate simulation requires a batter to score 88 runs at a strike rate of 173, whereby 60 runs are scored through boundaries.

**Table 3.** Ball-by-ball simulation of a Twenty20 ‘low’ strike rate innings (50 runs ± 1SD).

			Run Requirements	
Phase	Over No.	On-strike delivery No.	On-strike	Off-strike
PowerPlay	1	1, 4, 6	0, 0, 0	0, 1, 0
	2	1, 3, 5, 6	4, 0, 0, 0	0, 3
	3	2, 3, 4, 5	0, 0, 4, 0	0, 1
	4	1, 4, 6	0, 0, 1	4, 0, 0
	5	2, 4, 6	0, 0, 2	0, 0, 0
	6	1, 3, 6	1, 0, 0	1, 4, 1
Middle	7	1, 2, 3, 4	1, 1, 0, 0	4, 0
	8	1, 2, 4	1, 1, 1	4, 1, 1
	9	1, 2, 4, 6	1, 0, 2, 4	0, 1
	10	2, 4, 6	1, 1, 4	4, 1, 0
	11	1, 3, 4, 6	2, 6, 1, 2	1, 0
Close Innings	12	2, 3, 6	0, 1, 1	0, 0, 0
	13	2, 4, 6	2, 1, 1	1, 0, 0
	14	1, 5, 6	1, 1, 1	1, 4, 0
	15	3, 4, 6	1, 4, 6	1, 2, 0
	16	2	0	1, 2, 1

Ball-by-ball audio dictation provided in supplementary material A.

**Table 4.** Ball-by-ball simulation of a Twenty20 ‘high’ strike rate innings (100 runs ± 1SD).

			Run Requirements	
Phase	Over No.	On-strike delivery No.	On-strike	Off-strike
PowerPlay	1	1, 3, 5	0, 0, 4	0, 1, 0
	2	1, 3, 4,	1, 4, 0	0, 3, 0
	3	2, 3, 4, 6	0, 0, 1, 1	1, 4,
	4	1, 2, 3, 5	0, 4, 1, 4	0, 0
	5	1, 2, 5	1, 0, 0	0, 0, 0
	6	1, 4, 6	0, 1, 0	1, 4, 1
Middle	7	1, 5, 6,	6, 1, 0	4, 0, 4
	8	1, 3, 4	1, 1, 4	1, 1, 1
	9	1, 2	1, 1	1, 4, 1, 0
	10	1, 2, 3, 5, 6	1, 2, 1, 0, 1	1
	11	2, 3, 5	2, 1, 1	0, 0, 0
Close Innings	12	2, 3, 4, 6	2, 6, 4, 6	0, 1
	13	2, 4, 6	1, 6, 1	0, 0, 1
	14	2, 3, 5	1, 0, 1	4, 0, 1
	15	1, 3, 6	4, 2, 4	2, 0, 1
	16	1, 2	4, 0	2, 1

Ball-by-ball audio dictation provided in supplementary material B.

**Table 5.** Innings simulation summary and mean batting performance parameters from 10 experienced, top order batters.

Innings	‘low’ strike rate (50 runs ± 1SD)			‘high’ strike rate (100 runs ± 1SD)		
Phase	PowerPlay	Middle	Close Inn.	PowerPlay	Middle	Close Inn.
On-strike deliveries	20	18	13	20	16	15
Required strike rate	60	161	154	110	150	280
Scoring zone placements ^*^	3.5	3.1	1.2	3.3	2.5	2.9
Point deductions ^†^	2.3	1.6	0.9	2.5	1.4	0.8
Performance score ^§^ (%)	44.4	47.6	44.6	43.5	46.7	48.8

^*^ Intercepted deliveries which penetrate demarcated scoring zones - bat-ball contact score multiplied by two.

^†^ 1 point deducted for every failed attempt at ball contact.

^§^ Bat-ball contact scores tallied and adjusted for maximum available points (4 points per on-strike delivery).

In the half-century innings, batters achieved a batting performance score mean (SD): 72 (26) points, while the century produced a mean (SD): 88 (21). Table 5 presents by-phase summaries of both simulations and achieved performance scores adjusted to percent scores with associated point multipliers (scoring zone placement) and deductions (play-and-miss).

## 4 Discussion

This study has developed two comprehensive batting simulations which are unique to Twenty20 cricket. Descriptions of high and low strike rate individual innings which vary in strike rate and requirement of boundaries are detailed ball-by-ball. Additionally, we suggest a method of improving the ecological dynamics and validity of batting simulations. These considerations improve match realism allowing researchers to produce match representative data and are a tool for sport practitioners to bolster training programmes.

Previous studies have investigated the physiological costs of scoring an ODI century ( Goble & Christie, 2017; Houghton et al., 2011; Pote & Christie) or the effect of shuttle running volume ( Christie et al., 2008) on a batter’s physiology. However, the simulations developed in this study provide researchers with the necessary tools to investigate Twenty20 batting in a more comprehensive manner. Namely, the simulations developed in this study may highlight the impact of run scoring intensity (strike rate) on the batters’ physiology which previously was not possible. Additionally, compared to shuttle running protocols and net practice, batting simulations as described in this study provide a more accurate representation of the stressors imposed on a batter during match play ( Goble & Christie, 2017; Vickery et al., 2018).

Current theories of skill acquisition also attest to the benefit of utilizing realistic game scenarios in training. Reilly and colleagues (2009) pioneered the Specific Adaptation to Imposed Demands (SAID) principle, which suggests adaptations are specific to the stressors/demands placed on the athlete. Therefore, sport practitioners should prioritize training scenarios where the replication of match stressors is at the forefront. Recently, ecological dynamics frameworks have been described to guide practitioners in constructing highly specific game scenarios for high-level sport development ( Woods et al., 2020). In cricket, batting sessions which utilize SAID and ecological dynamics principles, like the batting simulations described in this study, may provide an ecologically valid tool to improve skill acquisition. Batters can routinely experience match-like fatigue and run scoring pressures which in turn may improve the athletes’ ability to cope once similar stressors arise during match play.

Batting simulations dictate run requirements thus, losing the metric of runs scored as a valuable measure of performance and incentive. To account for this, we implemented a method which objectively quantifies the batter’s performance from bat-ball contacts, stroke placement and dismissals. Batters achieved similar scores in both simulations ( Table 5) which may indicate a ceiling of skill set for our sample at the time of investigating or a lack of sensitivity in our scoring method. Future research should identify additional variables which may contribute to batting performance scores that are representative of skill. Further developing a dependable method of scoring batting performance will benefit both researchers and coaches. Sport practitioners can also adjust details of the Twenty20 simulations such as, the number and placement of scoring zones or setting batting performance score targets to create varying demands of difficulty for different athletes. Innings simulations, like BATEX ( Houghton et al., 2011) and those described in our study are naturally limited in flexibility. As a result, effectiveness for skill acquisition and the batter’s interest may reduce with repetition. To alleviate this, additional simulations which highlight unique game dynamics namely, recovering from a sudden loss of multiple wickets or catering to specific batting order positions could be developed.

## Conclusion

Batting research in cricket requires intricate methods of replicating the stressors and demands of an individual innings. By doing so, the physiological effects of batting on the batter may be investigated while also providing a tool for specific skill acquisition. The simulations developed in this study replicate an hour-long partnership consisting of 51 on-strike deliveries and played at either a high or low strike rate. Batters produced similar scores in both the high and low strike rate innings when quantifying their performance based on bat-ball contacts and stroke placement. Utilizing these simulations should provide an enhanced training stimulus to traditional net-based practice while future research investigates other factors that contribute to a batters performance.

## Data availability

Zenodo: Cricket-Research: Developing Twenty20 batting simulations. http://doi.org/10.5281/zenodo.4740316 ( Lopes, 2021)

This project contains the following underlying data:

• Supp.Material.A.m4a (audio description used to implement the low strike rate innings)
• Supp.Material.B.m4a (audio description used to implement the high strike rate innings)
• T20Simulation.R (Code to reduce ball-by-ball match data for development of additional batting simulations)
• T20TOP7.csv (Innings summaries produced by specialist batters in the Indian Premier League and international competition between 2006-2018)

Data are available under the terms of the Creative Commons Attribution 4.0 International license (CC-BY 4.0).
