# Supplementary material for: Novel Twenty20 batting simulations: a strategy for research and improved practice
Source: F1000Res. 2024 Feb 27;10:411. Originally published 2021 May 21. [Version 2] doi: 10.12688/f1000research.52783.2 (PMC11021879; doi:10.12688/f1000research.52783.2)
Supplement: Peer review history

# Reviewer report: Reviewer response for version 2

**DOI:** 10.5256/f1000research.121763.r262236

**Published:** 2024-04-23

**Authors:** Will Vickery

**Affiliations:** 1 Coaching and Officiating, Sport and Community Capability, Australian Sports Commission, Melbourne, Victoria, Australia

***General Comments :***

 The authors have provided a novel training methodology that many practitioners may find useful in their journey to develop and improve their T20 batters. With research existing in this area, it was good to see an evolution of this and how it has been able to help develop the protocols used in the current study. Below are some points for consideration, first general comments followed by more specific ones. The key points that need to be considered include:

- The representativeness of the batting simulations within batting context of T20 cricket (i.e. various batting positions and their role).
- Greater detail regarding the creation of and running of the batting simulations.
- Greater discussion of the findings from this study based on the aim.

*Specific Comments*

 Introduction Page 3: Consider rewording these sentences as the beginning of the second is an extension of the first but it begins with And, therefore not following grammatical standards. “In cricket, the availability of competitive match data enables the development of detailed innings simulations that can be an effective addition to practice programmes. And a reliable method of recreating the physiological baseline of competitive batting for further scientific inquiry.”

 Introduction Page 3: Please include the original occurrence of The Battlezone in the literature here “The Battlezone protocol mimics a small-sided cricket match reduced to eight players and six repeated bouts, each consisting of eight overs.” Renshaw, I., Chappell, G., Fitzgerald, D., Davison, J., & McFadyen, B. (2010, June). The battle zone: constraint-led coaching in action. In 1 of 1-Conference of Science, Medicine & Coaching in Cricket 2010 (p. 181).[Ref 2].

 Please also note that this is only one way in which The Battlezone was used, there were changes in the number of overs per batting group within other publications, along with changes to the field dimensions, field settings and rules. It should be noted in the current study that this is only an example of one Battlezone protocol.

 Introduction Page 3: It is unclear why the authors have decided to create two batting simulations based on the information presented within the Introduction. Some justification for why is required before stating that this is the aim.

 Methods Retrospective data acquisition - innings summaries Page 4: The authors chose to include international matches and the IPL. Although a high standard of cricket, justification is required as to why a domestic franchised tournament (IPL) was chosen alongside international standard matches which would arguably be considered the highest level of T20 cricket. Further, even with the high standard of the IPL potentially matching the international matches, justification for why it was chosen and not included other domestic franchise tournaments is needed.

 Methods Retrospective data acquisition - innings summaries Page 4: Please provide reasoning as to why “top order” was considered as someone within the first 7 batters when typically, those below batting position 3 are considered middle order. This is important since the length of a batting innings is likely to be considerably impacted based on batting position in a T20 innings where those at the top are more likely to have greater opportunity to bat for extended periods of time compared to those lower down the order. This potentially influences the runs scored and the strike rates of these batters.

 Methods Retrospective data acquisition - innings summaries Page 4: The authors state “To obtain innings summaries from the IPL, match scorecards were opened manually to import summaries produced the by top seven batters from both teams.”, was this this also the case for the international matches or was another method used?

 Methods Retrospective data acquisition - innings summaries Page 4:  It would be of interest to know the batting position that this number of deliveries most occurred “The resultant overlap occurred at 51 deliveries faced, with a total of 27 half-centuries (‘low’) and 18 centuries (‘high’).” Since this may provide greater context as to how the simulation is representative of the match situation where this is occurring which may have application for coaches wanting to implement this into their training.

 Methods Simulated innings task design Page 6: Were the international matches included in this part of the data collection process “Time motion analysis of Vivo IPL Twenty20 matches…”?

 Methods Simulated innings task design Page 6: Please include who this audio recording was intended for “An audio recording was created using a written script and computer text-speech function (Supplementary Material A, B) to govern intervals between overs/deliveries.”

 Methods Simulated innings task design Page 6: Were the batters required to keep track of the on-strike deliveries and run type themselves or were they assisted to make sure that all types and shuttles were completed? Please include more detail of how the batters ensured they “scored” the required amount of runs and completed the shuttle runs.  

 Methods Simulated innings task design Page 6: When describing the batting protocol, what happens if no bat-ball contact is made with regards to the shuttle running?

 Methods Simulated innings task design Page 6: Greater detail of what the scoring zone is and how it was used and modified throughout the situations is required. There is currently limited information about what it is and how it was used before this is mentioned here.

 Methods Simulated innings task design Page 6/7: Please provide reasoning for why “Boundaries will require one and a half shuttles for a ‘four’”. Is this common within the current T20 context?

 Methods Simulated innings task design Page 7: Was the running intensity monitored to ensure that batters did run at maximum intensity during each shuttle? Further, how was it determined what maximum intensity was prior to the batting simulation?

 Methods Simulated innings task design Page 7: The use of phases to represent the batting innings may not be representative of all batting innings that align with the strike rate/scoring that was used. Those batting in the lower batting position (e.g. 6, 7) are unlikely to bat during the initial power play overs (for example). Therefore, would this phase be relevant for those from these batting positions? This may need to be addressed when explaining a T20 batting innings within the introduction.

 Methods Simulated innings task design Page 8: With the simulation based on data from professional level cricket players, please justify why amateur players were used for this study. It is possible that the simulations do not match the playing conditions and scenarios experienced by the players in the current study.

 Discussion Page 10: Rather than “ecological dynamics” the term “representative” would be more suitable for describing a method of improving the batting simulations.

 Discussion Page 10: Consider including some of the findings from Ref [1]. Journal of Sports Sciences, 40(5), 498-508 and Lascu, A., Spratford, W., Pyne, D. B., & Etxebarria, N. (2021). Evaluating task design for skill development in an amateur female cricket team. Physical Education and Sport Pedagogy, 26(4), 330-344 to assist in explain how the representative of a task can be improved specifically in a cricket context.

 Discussion Page 10: Please change to wording of how BATEX is described as it was performed in a netted area rather than on a centre wicket “centre-wicket simulations, like BATEX..”.

 Discussion Page 10: When addressing “the simulations in this study critically lack a component of representative design due to the use of a projectile bowling machine.”, please include how the environment could be more representative of match context (i.e. the current study used an indoor, artificial surface with limited information gained from what would be happening in the field).

 Discussion Page 10-11: Much of the discussion focuses on how the study could be improved and become more representative of a match context. This is well developed and provides insight into what future research could include, however, there is limited discussion of the findings of the study when referring back to the aim. Consider including more about what aspects of the simulations did “mimic the demands of competitive match play”.

Is the rationale for developing the new method (or application) clearly explained?

Partly

Is the description of the method technically sound?

Partly

Are the conclusions about the method and its performance adequately supported by the findings presented in the article?

Partly

If any results are presented, are all the source data underlying the results available to ensure full reproducibility?

Yes

Are sufficient details provided to allow replication of the method development and its use by others?

Partly

Reviewer Expertise:

Sport coaching, cricket, practice design

I confirm that I have read this submission and believe that I have an appropriate level of expertise to confirm that it is of an acceptable scientific standard, however I have significant reservations, as outlined above.

## References

[1] : Evaluating task design for skill development in an amateur female cricket team. *Physical Education and Sport Pedagogy* .2021;26(4) : 10.1080/17408989.2020.1752648 330-344 10.1080/17408989.2020.1752648

[2] : The battle zone: Constraint-led coaching in action. *In Portus, M (Ed.) Proceedings of the 2010 Conference of Science, Medicine and Coaching in Cricket. Cricket Australia* .2010;

# Reviewer report: Reviewer response for version 2

**DOI:** 10.5256/f1000research.121763.r262240

**Published:** 2024-04-16

**Authors:** Farjana Akter Boby

**Affiliations:** 1 Department of Physical Education and Sports Science, Daffodil International University, Dhaka, Dhaka Division, Bangladesh

I would like to express my gratitude for the opportunity to review the interesting study titled "Novel Twenty20 batting simulations: a strategy for research and improved practice." It was a privilege to evaluate such a well-conducted study that addresses the need for realistic practice scenarios in cricket training. The study presents a thorough investigation into the development of batting simulations, providing valuable insights into the methods used and the potential applications of the simulations in cricket research and practice. The rigorous methodology and comprehensive description of the simulations contribute to the quality and relevance of the study.

 I commend the authors for their meticulous approach and attention to detail throughout the manuscript. The study's findings have the potential to significantly impact the field of cricket training and performance assessment.

**Strengths:**

 Rigorous Methodology: The authors have employed a systematic approach to develop the batting simulations, utilizing a combination of match data analysis, literature review, and pilot testing. The incorporation of both retrospective data acquisition and real-world testing enhances the validity and reliability of the simulations.

 Comprehensive Description: The manuscript provides a comprehensive overview of the batting simulations, including detailed descriptions of the scoring mechanisms, task design, and performance evaluation. This level of detail allows readers to understand the nuances of the simulations and their potential applications in cricket training and research.

 Ecological Validity: The simulations aim to replicate the physiological and cognitive demands of Twenty20 cricket, addressing the limitations of traditional net-based practice. By incorporating factors such as run scoring intensity, partnership dynamics, and match pressure, the simulations provide a more realistic training environment for batters.

**Suggestions for improvement:**

**Clarity in the Data Acquisition Section:** While the manuscript outlines the process of data acquisition, there is a lack of clarity regarding the selection criteria for the innings used in the study. Providing more explicit details on how specific innings were chosen from the ESPN Cricinfo database and the criteria used to narrow down the sample would enhance the transparency and reproducibility of the study.

**Discussion of Variability in Pilot Testing Results:** Although the results section mentions the performance scores achieved by participants in pilot testing, there is limited discussion on the variability observed among participants. Including a discussion on the range of performance scores, potential factors contributing to variability (e.g., individual differences in skill level, familiarity with the task), and implications for interpreting the results would provide valuable insights.

**Ecological Validity Considerations: **While the manuscript addresses the need for ecological validity in batting simulations, there is limited discussion on the extent to which the developed simulations capture the complexity of real match situations. Consideration of additional contextual factors that may influence batting performance in Twenty20 cricket (e.g., match conditions, opposition tactics) and their integration into the simulations would strengthen the ecological validity and practical relevance of the study.

**Generalization of Findings:** The study primarily focuses on the development and initial testing of the batting simulations with a specific group of participants. However, there is limited discussion on the generalizability of the findings to broader populations of cricket players, including those at different skill levels or age groups. Discussing the potential limitations and applicability of the simulations across diverse cricket contexts would enhance the relevance and utility of the study for a wider audience.

**Future Research Directions: **While the manuscript briefly mentions potential future research directions, such as exploring additional factors influencing batting performance, there is limited elaboration on specific avenues for further investigation. Providing more detailed suggestions for future research, including potential hypotheses to test and methodological considerations, would guide the development of follow-up studies and advance the field.

Is the rationale for developing the new method (or application) clearly explained?

Yes

Is the description of the method technically sound?

Yes

Are the conclusions about the method and its performance adequately supported by the findings presented in the article?

Yes

If any results are presented, are all the source data underlying the results available to ensure full reproducibility?

No source data required

Are sufficient details provided to allow replication of the method development and its use by others?

Yes

Reviewer Expertise:

Sports ScienceExercise ScienceSports InjuriesExercise PerformanceSport BiomechanicsExercise Physiology

I confirm that I have read this submission and believe that I have an appropriate level of expertise to confirm that it is of an acceptable scientific standard, however I have significant reservations, as outlined above.

# Reviewer report: Reviewer response for version 2

**DOI:** 10.5256/f1000research.121763.r250661

**Published:** 2024-04-12

**Authors:** Sean Muller

**Affiliations:** 1 Centre for Smart Analytics, Federation University Australia, Ballarat, Victoria, Australia

Thank you for your revisions and stating limitations of the project.

Is the rationale for developing the new method (or application) clearly explained?

Partly

Is the description of the method technically sound?

Yes

Are the conclusions about the method and its performance adequately supported by the findings presented in the article?

Yes

If any results are presented, are all the source data underlying the results available to ensure full reproducibility?

No source data required

Are sufficient details provided to allow replication of the method development and its use by others?

Yes

Reviewer Expertise:

Expertise and motor skill learning.

I confirm that I have read this submission and believe that I have an appropriate level of expertise to confirm that it is of an acceptable scientific standard.

# Reviewer report: Reviewer response for version 1

**DOI:** 10.5256/f1000research.56104.r85930

**Published:** 2021-06-15

**Authors:** Habib Noorbhai

**Affiliations:** 1 Department of Sport & Movement Studies, Faculty of Health Sciences, University of Johannesburg, Johannesburg, South Africa

Thank you very much for the opportunity to review this interesting study, which would be a valuable contribution to the cricket sciences literature.

 Below, I have suggested a few points that will assist in improving the standard of the paper:

1. It is not clear why a bowling machine was utilised? Bowling / real-life kinematics are key, especially for high strike rate scoring. This needs to be listed as a limitation.
2. Were the deliveries bowled permutated?
3. The discussion requires a well-rounded discussion including aspects of task and temporal constrains of batting, motor control, performance analysis and batting kinetics/kinematics (even though this study is based on simulation and research strategy).

You may also want to consider other T20 studies your paper:

1. Davis, J., Perera, H. and Swartz, T.B., 2015. A simulator for Twenty20 cricket** ^1^
2. Sholto-Douglas, R., Cook, R., Wilkie, M. and Christie, C.J.A., 2020. Movement demands of an elite cricket team during the big bash league in Australia ^2^
3. Noorbhai, H. (2020). The backlift techniques of cricketers in the Indian Premier League: batting implications for the shorter format. International Sports Studies ^3^
4. Irvine, S. and Kennedy, R., 2017. Analysis of performance indicators that most significantly affect International Twenty20 cricket ^4^

Is the rationale for developing the new method (or application) clearly explained?

Yes

Is the description of the method technically sound?

Yes

Are the conclusions about the method and its performance adequately supported by the findings presented in the article?

Yes

If any results are presented, are all the source data underlying the results available to ensure full reproducibility?

No source data required

Are sufficient details provided to allow replication of the method development and its use by others?

Yes

Reviewer Expertise:

Cricket Sciences and Sports Biomechanics.

I confirm that I have read this submission and believe that I have an appropriate level of expertise to confirm that it is of an acceptable scientific standard, however I have significant reservations, as outlined above.

## References

[1] : A Simulator for Twenty20 Cricket. *Australian & New Zealand Journal of Statistics* .2015;57(1) : 10.1111/anzs.12109 55-71 10.1111/anzs.12109

[2] : Movement Demands of an Elite Cricket Team During the Big Bash League in Australia. *J Sports Sci Med* .19(1) :59-64 32132828 PMC7039030

[3] : The backlift techniques of cricketers in the Indian Premier League: batting implications for the shorter format. *International Sports Studies* .2020;42(1) : 10.30819/iss.42-1.05 47-54 10.30819/iss.42-1.05

[4] : Analysis of performance indicators that most significantly affect International Twenty20 cricket. *International Journal of Performance Analysis in Sport* .2017;17(3) : 10.1080/24748668.2017.1343989 350-359 10.1080/24748668.2017.1343989

# Response

**Published:** 2024-02-22

**Authors:** Tiago Lopes

**Affiliations:** University of the Witwatersrand, South Africa

Thank you providing input to improve the quality of this work. Following your comments, the manuscript has been adjusted to discuss the limitations of using a bowling machine in research and batting simulations. A clarification on the set-up of the bowling machine and permutation of deliveries is provided. Reviewed literature has also been updated from your input. For brevity, some information has remained omitted including the T20 simulator by Davis, Perera & Swartz which addresses a different objective to that presented in this study.

# Reviewer report: Reviewer response for version 1

**DOI:** 10.5256/f1000research.56104.r85928

**Published:** 2021-06-09

**Authors:** Sean Muller

**Affiliations:** 1 Centre for Smart Analytics, Federation University Australia, Ballarat, Victoria, Australia

Thanks for the opportunity to review an interesting study. Well done to the authors for going to great effort to design and test the simulated batting task. I only have a few points to make, which the authors should consider and revise the manuscript. First, I understand the reasoning for using a bowling machine, but it does not present important bowler kinematic cues. Contextual (field-placing) information is also important particularly for T20 cricket. Was the complete field placing presented to batsmen throughout phases of the test? These cues are important to load the cognition of batters like occurs in competition (noting the authors have mentioned  “…to closely replicate match-play….”). Presenting these sources of information is important for future design of simulators. Please discuss this as a limitation and how it might be improved upon in future research. Second, the correct terminology is representative task design, not ecological validity (see Araújo & Davids, 2015 ^1^). Third, introduction and discussion sections; ecological-dynamics theory is not the only framework that guides “….constructing highly-specific game scenarios….”. The specificity of practice hypothesis (see Magill & Anderson, 2017 ^2^; Thorndike, 1906 ^3^) provides the same guidance. Please revise this in the manuscript to provide a more balanced discussion of the literature.

Is the rationale for developing the new method (or application) clearly explained?

Partly

Is the description of the method technically sound?

Yes

Are the conclusions about the method and its performance adequately supported by the findings presented in the article?

Yes

If any results are presented, are all the source data underlying the results available to ensure full reproducibility?

No source data required

Are sufficient details provided to allow replication of the method development and its use by others?

Yes

Reviewer Expertise:

Expertise and motor skill learning.

I confirm that I have read this submission and believe that I have an appropriate level of expertise to confirm that it is of an acceptable scientific standard, however I have significant reservations, as outlined above.

## References

[1] : Towards a theoretically-driven model of correspondence between behaviours in one context to another: Implications for studying sport performance. *International Journal of Sport Psychology* .2015;46:745-757

[2] : Motor learning and control: Concepts and applications (11th ed.). *McGraw Hill* .2017;

[3] : Principles of Teaching. *Seiler* .1906;

# Response

**Published:** 2024-02-22

**Authors:** Tiago Lopes

**Affiliations:** University of the Witwatersrand, South Africa

Thank you for your valuable input and fair critique to this version of the manuscript. Following your comments the manuscript has been revised to include; a more balanced discussion of representative task design with updated references, critique around the use of bowling machines in future studies and simulators which will disregard kinematic cues of the bowling action, clarification on the presentation and instruction provided to batters for fielding positions.
